# Exploring the associations between elevated plasma SP-D levels and OSCAR gene expression as potential biomarkers in patients with COPD: a cross-sectional study

**DOI:** 10.3389/fphar.2024.1376394

**Published:** 2024-07-18

**Authors:** Saima Mukhtar, Nakhshab Choudhry, Saqib Saeed, Asif Hanif, Aamir J. Gondal, Nighat Yasmin

**Affiliations:** ^1^ Department of Physiology, Rahbar Medical and Dental College, Lahore, Pakistan; ^2^ Department of Biomedical Sciences, King Edward Medical University, Lahore, Pakistan; ^3^ Department of Biochemistry, King Edward Medical University, Lahore, Pakistan; ^4^ Institute of TB and Chest Medicine, Mayo Hospital, Lahore, Pakistan

**Keywords:** chronic obstructive pulmonary disease, osteoclast-associated receptor, lung function test, surfactant protein D, gene expression

## Abstract

**Background:** Chronic obstructive pulmonary disease (COPD) imposes a substantial burden on patients and healthcare systems. Spirometry is the most widely used test to diagnose the disease; however, a surrogate marker is required to predict the disease pattern and progression.

**Objectives:** The aim of the current study was to explore the association of elevated levels of plasma surfactant protein D (SP-D) with gene expression of osteoclast-associated receptor (OSCAR) and lung functions as potential diagnostic biomarkers of COPD.

**Methods:** This cross-sectional study employed convenience sampling. As men compose the majority of patients in the outpatient department and with smoking being common among Pakistani men, choosing men offered a representative sample. Using a post-bronchodilator forced expiratory volume in the first second (FEV1) to a forced vital capacity (FVC) of less than 0.70 (FEV1/FVC <0.7), COPD patients were diagnosed on spirometry (n = 41). Controls were healthy individuals with FEV1/FVC >0.7 (n = 41). Plasma SP-D levels were measured using an enzyme-linked immunosorbent assay (ELISA). The gene expression of OSCAR was determined by real-time polymerase chain reaction (qPCR) and subsequently analyzed by the threshold cycle (Ct) method. Statistical Package for Social Sciences (SPSS) version 20 was used for statistical analysis.

**Results:** The mean BMI of controls (25.66 ± 4.17 kg/m^2^) was higher than that of cases (23.49 ± 2.94 kg/m^2^ (*p* = .008)). The median age of controls was 49 years (interquartile range (IQR) 42.0–65.0 years) and that of cases was 65 years (IQR = 57.50–68.50). SP-D concentration was not significantly higher in COPD patients [4.96 ng/mL (IQR 3.26–7.96)] as compared to controls [3.64 ng/mL (IQR 2.60–8.59)] (*p* = .209). The forced expiratory ratio (FEV1/FVC) and FEV1 were related to gene expression of OSCAR (*p* = <.001). The gene expression of OSCAR was significantly related to SP-D (*p* = .034). A multiple regression model found FEV1 and FVC to have a significant effect on the gene expression of OSCAR (*p*-values <0.001 and 0.001, respectively).

**Conclusion:** Gene expression of OSCAR was increased in COPD patients and related directly to SP-D levels and inversely to lung functions in cohort of this study, suggesting that OSCAR along with SP-D may serve as a diagnostic biomarker of COPD.

## 1 Introduction

Chronic obstructive pulmonary disease (COPD) is a chronic respiratory syndrome characterized by symptoms such as cough, sputum production, and breathlessness. This syndrome results in respiratory symptoms like inflammation, mucus hypersecretion in the airways, and limited airflow (Bartolomé and Celli, 2018). COPD is mostly observed in smokers and people exposed to noxious gases (NICE, 2022). It is an under-diagnosed disease globally due to a lack of knowledge about its cause(s), prevalence, and progression. COPD has become an ongoing epidemic in both developed and underdeveloped countries, causing 3.23 million deaths in 2019, making it the third leading cause of mortality globally (World Health Organization, 2023). Pakistan has a high COPD prevalence of 18.5% among its population aged 40 years and above (Jarhyan et al., 2022).

COPD causes an enhanced inflammatory response that damages lung tissues and manifests systemic comorbidities such as cardiovascular damage, musculoskeletal dysfunction, osteoporosis, anemia, gastrointestinal symptoms, neuronal damage, and behavioral effects (Cordova-Rivera et al., 2019). The disease progression is marked by episodes of exacerbation characterized by acute aggravation of symptoms of dyspnea, sputum volume, and purulence. The progression results in compromised quality of life and requires frequent hospital visits or admissions (Kjaergaard et al., 2020) Specific biomarkers are required for its early detection, diagnosis, treatment, and drug development. Surfactant protein D (SP-D), a glycoprotein of the collectin family, is secreted by type II alveolar cells. SP-D causes opsonization, neutralization, and agglutination of pathogens by phagocytes (Liao et al., 2021). It has been extensively studied as a diagnostic biomarker for COPD. SP-D concentration increases in plasma due to its transmigration from the alveolar compartment into systemic circulation following smoke exposure. Studies indicate that the average level of SP-D in COPD patients is significantly higher than that in normal controls (Dalgard et al., 2021).

Increasing inflammation, lung parenchymal injury, and leaky capillaries facilitate the shift of SP-D from the pulmonary milieu to systemic circulation in COPD (Tiwari et al., 2024). This SP-D dysregulation and deficiency lead to hampered clearance of apoptotic and necrotic tissue and inflammation flare-up in the lung tissue, worsening the disease (Arroyo et al., 2018).

Recent evidence hints at the role of SP-D in activating circulating monocytes to become tissue macrophages by interacting with the osteoclast-associated receptor (OSCAR). OSCAR is an immunoglobulin-G (IgG)-like activating receptor for collagen and modulates the innate and adaptive responses by enhancing proliferation and maturation of immune cells (Elango et al., 2022). It has been observed that OSCAR is enhanced in the peripheral blood monocytes of patients with chronic inflammatory conditions like rheumatoid arthritis (Jung et al., 2019). Interestingly, SP-D is a ligand for OSCAR, and their interaction (OSCAR-SP-D interaction) triggers TNF-α production by inflammatory monocytes. The circulating monocytes exposed to leaked SP-D then release TNF-α and become mature alveolar macrophages. The increased alveolar macrophage infiltration initiates a chronic inflammatory response, leading to tissue destruction and alveolar damage (Schultz et al., 2015; Sorensen, 2018).

The current study aims to evaluate the expression of OSCAR in blood leukocytes in relation to SP-D levels and lung function in COPD patients. Blood was collected from COPD patients after performing spirometry under sterilized conditions. Plasma was isolated to determine SP-D levels using ELISA. RNA was isolated from plasma to synthesize cDNA, and the expression of OSCAR was quantified with the fold-change method. Results indicated that OSCAR expression was higher in the white blood cells of COPD patients than in smoker controls, was positively associated with circulating SP-D levels, and was negatively associated with lung functions, indicating a relation between OSCAR and chronic inflammation and deteriorating lung function.

Anything that can prevent the exacerbations or even predict future progression of the disease would be pivotal in the management of COPD. The current study will help open new avenues for rapid detection, diagnosis, and possible development of novel biomarkers for disease severity. Once proven, this would help foster advancement toward benefiting the community by reducing the burden of disease.

## 2 Methods

### 2.1 Subjects

From November 2021 to May 2022, 82 patients were enrolled from the Outpatient Department of Chest Medicine, Mayo Hospital, Lahore (Shein-Chung Chow et al., 2018). This was a cross-sectional study in which non-probability convenience sampling was adopted to enroll patients. The research was approved by the Institutional Review Board (IRB), Ethical Committee, and Advanced Studies and Research Board (AS&RB) of King Edward Medical University/Mayo Hospital, Lahore.

In this study, 41 patients were enrolled based on history (including chronic cough, sputum production, and breathlessness), examination, and spirometry. Those with a post-bronchodilator ratio (after the administration of 200 mcg salbutamol) of less than 0.70 (FEV1/FVC <0.70) were labeled as having COPD. Post-bronchodilator response was considered significant if there was a 12% increase from baseline and/or a 200-mL improvement in FEV₁, and the patient was excluded (Bhatt et al., 2022). Due to the overlap of symptoms and disease processes between COPD and asthma, the study also excluded individuals with asthma or those having symptoms suggestive of bronchial hyper-reactivity and diagnosed cases of asthma COPD overlap syndrome (ACOS). Patients with active tuberculosis were not included owing to its association with changes in structural, inflammatory, and genetic expression independently of COPD. Rheumatoid arthritis (RA) and osteoarthritis (OA) patients were also excluded as the associated chronic systemic inflammation and medication could interfere with inflammatory biomarkers and genetic expression of OSCAR. Lastly, patients on oral steroid therapy were not included in the study as systemic steroids are known to influence gene expression, hence impacting the study outcomes. Healthy controls (n = 41) were recruited from the general population of Lahore and attendants of patients visiting the hospital. All patients were enrolled after obtaining informed consent. Matching was done in terms of gender, socioeconomic and smoking status, and ethnicity. Both cases and controls included Pakistani men aged between 35 and 76 years. As smoking is more frequent in men in Pakistan, where it is seen as a sign of masculinity and superiority, and is socially unacceptable for women, most COPD patients are men (Masud and Oyebode, 2018). The only female visiting the Chest Department was not selected due to the convenience sampling technique, resulting in an all-male cohort.

### 2.2 Spirometry

Height (centimeters), weight (kilograms), ethnic background, and smoking status of all participants (cases and controls) were recorded, and spirometry was performed on an electronic spirometer (MIR Spirodoc) (Italy, software version 2.6). In brief, to perform spirometry, patients were advised to fully inhale, seal their mouth around the sterile mouthpiece, and breathe out as hard and as much as possible for at least 6 sec. Cases inhaled 200 µg of salbutamol (Salbo, Getz Pharma, KIA, Karachi, Pakistan) via a spacer device after getting a first record. After a 15-min waiting period, the process was repeated until technically acceptable curves were obtained, and the best of three readings were considered to calculate lung functions (Sim et al., 2017).

### 2.3 Blood sampling and processing

To obtain a blood sample, venipuncture was conducted, and 5 mL of blood was drawn. Blood (2 mL and 3 mL) was transferred into two EDTA-containing tubes separately. Tubes were placed on ice and transported to the research laboratory within 4 h of collection for RNA isolation. The remaining 3 mL of blood was used to separate the plasma and perform ELISA for the detection of SP-D concentration.

### 2.4 Genetic expression

#### 2.4.1 RNA isolation

RNA was isolated using the commercially available GeneJET RNA Purification Kit according to the manufacturer’s instructions (Thermo Fisher Scientific). In brief, the collected blood sample stored at 4°C was used for RNA isolation. Whole blood (0.5 mL) was centrifuged at 400 g for 5 min at 4°C, and the clear supernatant (plasma) was discarded using a pipette to obtain cells as pellets. The cell pellet was re-suspended in 600 µL of lysis buffer (supplemented with β-mercaptoethanol) and mixed thoroughly using a vortex. Next, 450 µL of 96% ethanol was added and mixed using a vortex. A measure of 700 μL of the lysate was transferred to the GeneJET RNA Purification Column inserted in a collection tube and centrifuged for 1 min at ≥12,000 × g. The flow-through was discarded, and the purification column was placed back into the collection tube. The GeneJET RNA Purification Column was then placed into a new 2-mL collection tube, and 700 µL of Wash Buffer 1 (supplemented with ethanol) was added to it. The column was centrifuged for 1 min at ≥12,000 × g, and the flow-through was discarded. The purification column was placed back into the collection tube. Wash Buffer 2 (supplemented with ethanol) was added to the GeneJET RNA Purification Column twice, and each time it was centrifuged for 1 min at ≥12,000 × g, with the flow-through being discarded after each wash. Finally, 250 µL of Wash Buffer 2 was added to the column and centrifuged for 2 min at ≥12,000 × g. To elute RNA, 50 µL of nuclease-free water was added to the center of the GeneJET RNA Purification Column membrane, and the column was centrifuged for 1 min at ≥12,000 × g. Purified RNA was stored at −20°C until used for quantification and cDNA synthesis.

#### 2.4.2 cDNA synthesis

RNA (1 µg) was used for cDNA synthesis using the commercially available RevertAid First Strand cDNA Synthesis Kit (Thermo Fisher Scientific) as per the manufacturer’s instructions. In brief, 1 µg of purified RNA was taken in a PCR tube and mixed with buffer, random primers, dNTPs, and reverse transcriptase enzyme. Nuclease-free water was added to adjust the total reaction volume to 10 μL. A negative template control (NTC) was also included, which contained all components but no DNA to rule out any contamination or primer dimers.

The thermal cycling protocol consisted of a hot start at 95°C for 3 min. Then, 30 cycles were repeated as follows: 95°C for 40 sec, 52°C for 30 sec, and 72°C for 40 sec and heating at 72°C for 5 min and 4°C ∞ hold until further analysis.

#### 2.4.3 Real-time PCR

**FIGURE 1:**
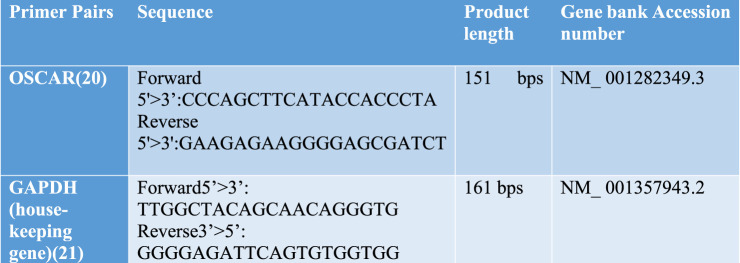
Forward and reverse primers for *OSCAR* and housekeeping gene glyceraldehyde 3-phosphate dehydrogenase (*GAPDH*).

cDNA was used for qRT-PCR using the SYBR green-based kit Maxima SYBR Green/ROX qPCR Master Mix (2X) (Thermo Fisher Scientific). Forward and reverse primers for qRT-PCR are shown in Figure 1. *GAPDH*, a housekeeping (HK) gene, was used as an internal control in the analysis. Real-time PCR was then performed in triplicate using the AriaMx Real-time PCR System. The master mix was prepared after thawing and gently vortexing all the solutions. The master mix contained the Maxima SYBR Green/ROX qPCR Master Mix (2X) = µL, forward primer = 0.5 µL, reverse primer = 0.5 µL, template DNA = 1 µg, and nuclease-free water to make a total volume of up to 12.5 µL. The master mix was dispensed into PCR tubes, mixed gently, and placed in a thermal cycler. cDNA (5 μL) was added to each tube. The thermal cycler was programmed for an initial denaturation at 95°C for 3 min (one cycle), followed by 40X cycles of denaturation at 95°C for 15 sec, annealing at 60°C for 30 sec, and extension at 72°C for 30 sec.

Melting curves were obtained, and threshold cycle (CT) values of both housekeeping genes were used to determine and calculate relative gene expression. The 2^−ΔΔCT^ method was used for interpretation as follows:

$$2^{-∆∆\text{CT}}=\left[\left(\text{CT gene of Interest}-\text{ CT internal control}\right)\text{ sample }A-\;\left(\text{CT gene of interest }–\text{ CT Internal control}\right)\text{ sample }B)\right],$$

where internal control represents the HK gene, sample A represents the cases, and sample B represents the controls (Kuang et al., 2018).

### 2.5 ELISA

The Invitrogen human SP-D ELISA Kit (EH436RB) was used to detect SP-D levels in the plasma of the cohort. Standards, quality control, and samples were run in duplicates. Plasma samples were diluted two-fold using 1X assay diluent. Wash buffers, diluent, biotin conjugate, and streptavidin–HRP solution were prepared according to the manufacturer’s instructions. Standards were serially diluted for the standard curve. Samples and standards were added to the wells after thawing, and ELISA plates were incubated for 2.5 h at room temperature. After the addition of the biotin conjugate, the samples were incubated for another hour with gentle shaking. Streptavidin HRP was added to wells after discarding the previous solution and washing thrice with wash buffer. Incubation with gentle shaking was done for 45 min. The solution was discarded, and wells were washed three times with wash buffer. The TMB substrate was added and incubated for 30 min in the dark, after which stop solution was added and gently tapped. Color change was observed, and absorbance was measured at 450 nm using the ELISA Reader (Multiskan FC Microplate Photometer) within 30 min (Hayrapetyan et al., 2023).

## 3 Statistical analysis

The study data are expressed as the mean ± standard deviation and median (interquartile range, IQR) for continuous variables and as frequency for categorical variables. The Shapiro–Wilk test of normality suggested a non-normal distribution of continuous data. A non-parametric Mann–Whitney U test was used for independent group comparisons. The chi-square test was used for categorical variables like smoking status and time since the last cigarette. The Spearman rank correlation was applied to compute the relationship between OSCAR and SP-D and lung functions. Multiple linear regression was performed to quantify the relationship between SP-D and lung functions and OSCAR gene expression.

## 4 Results

The study included all men aged between 35 and 73 years. The median age of controls was 49 years (interquartile range (IQR) 42.0–65.0), and that of cases was 65 years with an IQR of 57.50–68.50.

Among the controls (n = 41), 27 were never smokers (65%), 3 were ex-smokers (7.3%), and 11 were current smokers (26.8%). The COPD group (n-41) had 2 never-smokers (4.9%), 19 ex-smokers (46.3%), and 20 current smokers (48.8%). The mean BMI of controls was 25.66 ± 4.17 kg/m^2^, and that of cases was 23.49 ± 2.94 kg/m^2^. There were 9 (21.9%), 14 (34%), and 18 (43.9%) controls belonging to the upper-middle, lower-middle, and upper-lower classes, respectively. A total of 5 (12.2%), 11 (26.8%), and 25 (60.9%) patients with COPD were from the upper-middle, lower-middle, and upper-lower classes, respectively Table 1.

**TABLE 1:** Demographic and physiological characteristics of study subjects.

Variable	Control (n = 41)	COPD (n = 41)	*p*-value
Age in years (median, IQR)	49 (42–65)	65 (57.5–68.5)	
BMI in kg/m^2^ (mean ± SD)	25.66 (±4.17)	23.49 (±2.94)	0.008*
Socioeconomic status^@^ n (%)			
Upper middle (16–25)	9 (21.9%)	5 (12.2%)	
Lower middle (11–15)	14 (34%)	11 (26.8%)	
Upper lower (5–10)	18 (43.9%)	25 (60.9%)	
Smoking status n (%)			
Never smoker	28 (68.29%)	2 (4.88%)	
Ex-smoker	3 (7.32%)	19 (46.34%)	
Current smoker	10 (24.39%)	20 (48.78%)	
Pack years of smoking (median, IQR)	0 (0–4.1)	22.5 (10–40)	<.001§
Time since the last cigarette in years n (%)			
Current smoker	10 (24.39%)	20 (48.78%)	
Less than 1 year	0 (0%)	5 (12.20%)	
Less than 10 years	01 (2.44%)	8 (19.51%)	
More than 10 years	02 (4.88%)	6 (14.63%)	
Non smoker	28 (68.29%)	2 (4.88%)	
Close family member affected n (%)			
Yes	5 (12.2%)	14 (34.1%)	<.001§
No	36 (87.8%)	27 (65.9%)	
SP-D levels in ng/mL (median, IQR)	3.64 (2.60–8.59)	4.96 (3.26–7.96)	0.209†
OSCAR gene expression in folds (median, IQR)	1.1 (0.15–4.50)	77.30 (24.85–180)	<.001†

BMI, body mass index; COPD, chronic obstructive pulmonary disease; SP-D, surfactant protein D; IQR, inter quartile range; OSCAR, osteoclast-associated receptor. ^@^Socioeconomic status calculated as per Modified Kuppuswamy’s scale (Wahid et al., 2022).

*Independent-sample *t*-test used; †Mann–Whitney U-test used; § chi-square applied. *p* = <0.05, significant and *p* = <0.001, highly significant. Values are presented as the mean ± SD or median IQR.

The average smoking history of controls was 0.0 (median) with an IQR of 0.0–4.1 pack years, while smokers had an average smoking history of 22.5 and an IQR of 10–40.0 median pack years. One pack year is defined as 20 cigarettes per day for 1 year.

Furthermore, 10 (24.39%) of controls and 20 (48.78%) of COPD patients were current smokers. No (0%) control and 5 (12.2%) of COPD patients were smoking for less than 1 year; 01 (2.4%) normal and 8 (19.5%) patients had smoked for less than 10 years; 02 (4.9%) of the control group and 6 (14.63%) of the patient cohort had smoked cigarettes for more than 10 years; 28 (68.29%) controls and 2 (4.88%) were non-smokers. A total of 5 (12.2%) controls and 14 (34.1%) cases had family members affected with COPD, while 36 (87.8%) controls and 27 (65.9%) cases did not have any close family member suffering from COPD (Table 1).

As shown in Table 1, no significant correlation (*p* = 0.209) was observed between higher surfactant protein D (SP-D) levels of 4.96 ng/mL (IQR 3.26–7.96) in COPD patients as compared to 3.64 ng/mL with an IQR of 2.60–8.59 in controls. The limited number of observations can account for at least some of this conclusion.

The forced expiratory ratio (FEV1/FVC) was 100.63% ± 6.65% of the expected value for the controls, while their forced expiratory volume in the first second (FEV1) was 97.88 ± 18.93 percent of the expected value. For the patients, FEV1/FVC was 63.67 ± 6.62 of the expected value, while their FEV1 was 50.78% ± 16.32% of the expected value. Expected values were estimated according to the age, body mass index, and ethnic background of the subjects. FEF2575 was 2.72 ± 0.95 in controls and 0.66 ± 0.33 in cases. FEV1, FVC, FEV1/FVC, and FEF2575 of controls were significantly different from those of cases (*p* = <.001 and 0.000). The lung functions were depressed in COPD patients as compared to healthy controls. Detailed lung functions are given in Table 2.

**TABLE 2:** Various lung functions in controls and COPD patients.

Variable	Control n = 41	COPD n = 41	*p*-value
FEV1 (L/sec) (mean ± SD)	2.85 ± 0.64	1.28 ± 0.46	<.001*
FEV1 %pred (mean ± SD)	97.88 ± 18.93	50.78 ± 16.32	<.001*
FVC (L/sec) (mean ± SD)	3.58 ± 0.76	2.47 ± 0.73	<.001*
FVC %pred (mean ± SD)	99.63 ± 19.75	77.98 ± 19.91	<.001*
FEV1/FVC (mean ± SD)	79.48 ± 5.51	49.16 ± 5.43	<.001*
FEV1/FVC %pred (mean ± SD)	100.63 ± 6.65	63.67 ± 6.62	<.001*
FEF2575 (L/sec) (mean ± SD)	2.72 ± 0.95	0.66 ± 0.33	0.000
FEF2575 %pred (mean ± SD)	80 ± 21.91	21.44 ± 9.48	0.000

COPD, chronic obstructive pulmonary disease; FEV1, forced expiratory volume at 1 s; %pred, percent of predicted value; FVC, forced vital capacity. FEF2575, forced expiratory flow at 25%–75%. Independent-sample *t*-test used. *p* = <0.05, significant. Values are presented as the mean ± SD.

Genetic expression of the *OSCAR* gene was 1.1 (0.15–4.50) in controls and significantly increased to 77.30 (24.85–180) in COPD patients with a *p*-value of <0.001.

**FIGURE 2:**
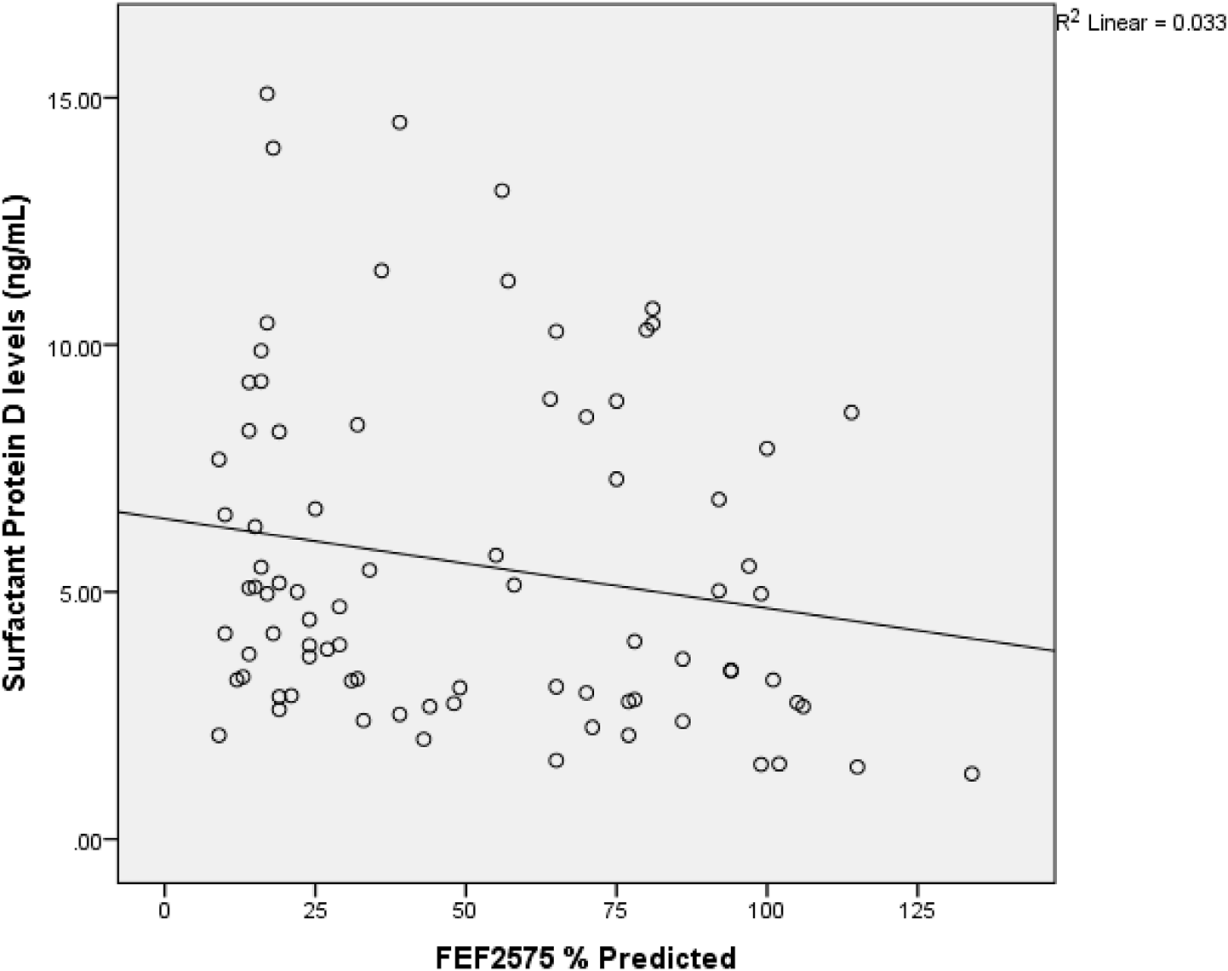
Spearman rank correlation between FEF2575% of predicted and surfactant protein D. r = −0.233 and *p* = 0.035 among the study subjects.

**FIGURE 3:**
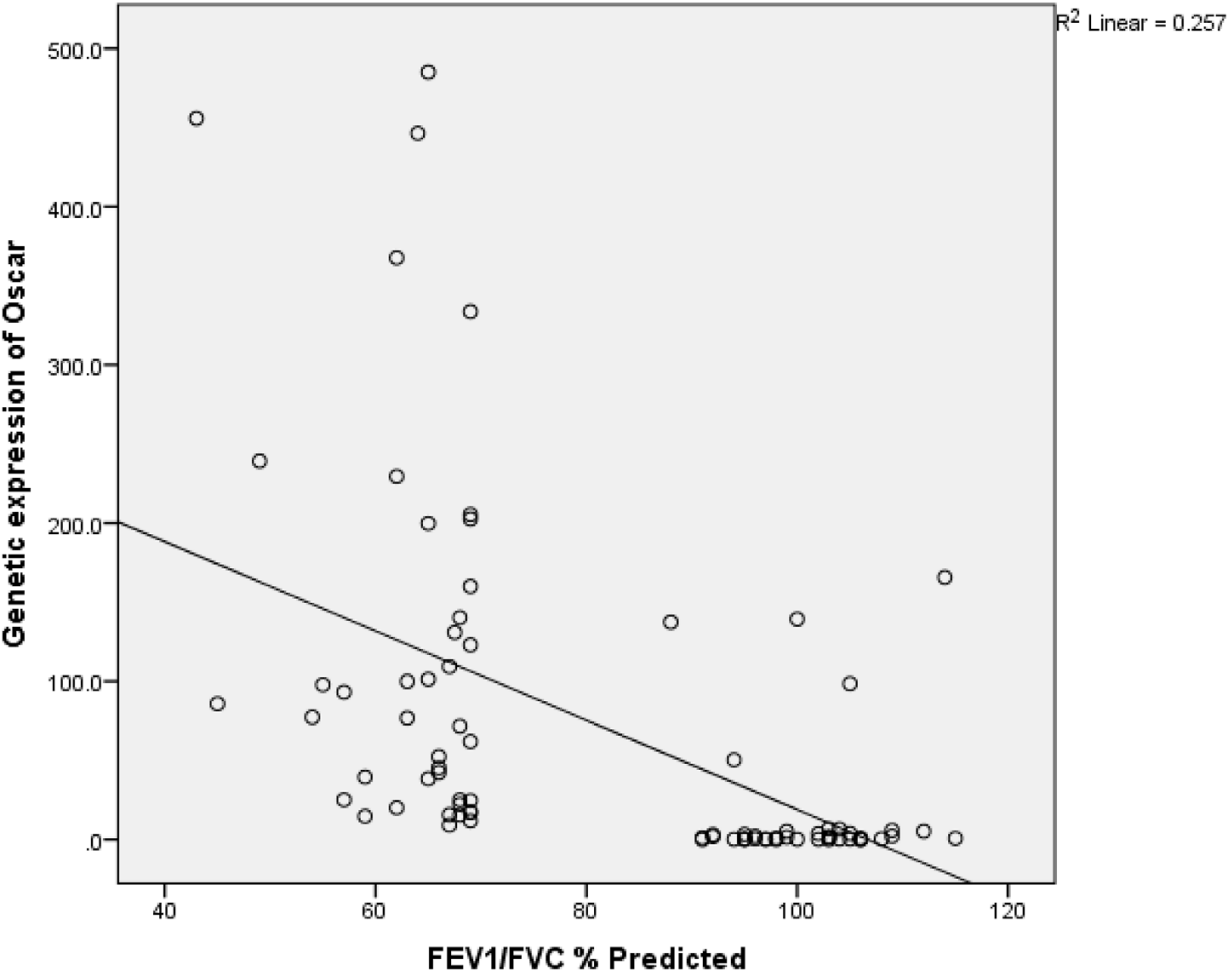
Spearman rank correlation between FEV1/FVC and *OSCAR* gene expression. r (79) = −0.645 and *p* = <0.001 among the study subjects.

A direct but weak relationship between *OSCAR* gene and surfactant protein D level (r = 0.234, *p* = .034) was found (Table 3). FEF2575% of predicted and surfactant protein D had a weak indirect relation (r = −0.233 and *p* = 0.035) among the study subjects as shown in Figure 2. Figure 3 shows an inverse and moderate relation between FEV1/FVC and OSCAR gene expression (r = −0.630 and *p* = .001).

**TABLE 3:** Spearman rank correlation of different variables with OSCAR gene expression.

Variable	Controls (n = 41)		Cases (n = 41)		Overall (n = 82)	
	OSCAR gene expression (r)	*p*-value	OSCAR gene expression (r)	*p*-value	OSCAR gene expression (r)	*p*-value
Age of the subject (years)	−0.127	0.429	−0.099	0.539	0.238	0.031*
Body mass index (kg/m^2^)	0.117	0.466	−0.090	0.574	−1.96	0.078
Pack years of smoking	0.046	0.776	−0.075	0.640	0.494	<0.001
Years since symptoms	-	-	0.019	0.907	0.682	<0.001
SP-D (ng/mL)	0.138	0.390	−0.039	0.811	0.234	0.034*
FEV1 (L/sec)	0.005	0.973	−0.033	0.836	−0.601	<0.001
FEV1% pred	0.091	0.573	−0.148	0.356	−0.60	<0.001
FVC (L/sec)	−0.029	0.857	0.025	0.875	−0.444	<0.001
FVC % pred	0.035	0.829	−0.006	0.972	−0.351	0.001
FEV1/FVC	0.169	0.290	−0.139	0.387	−0.630	<0.001
FEV1/FVC % pred	0.124	0.440	−0.188	0.240	−0.645	<0.001
FEF2575 (L/sec)	0.118	0.463	−0.038	0.815	−0.608	<0.001
FEF2575% pred	0.176	0.270	−0.042	0.794	−0.625	<0.001
Disease severity #	-	-	0.188	0.239	0.505	<0.001
COPD assessment test	-	-	−0.117	0.465	0.660	<0.001

FEV1, forced expiratory volume at 1 s; %pred, percent of predicted value; FVC, forced vital capacity; FEF2575, forced expiratory flow between 25% and 75% of vital capacity. Spearman rank correlation was used. *p* = <0.05, significant; *p* = <0.001, highly significant. *Disease severity according to the global strategy for the prevention, diagnosis, and management of COPD (GOLD) criteria (Augusti, 2018).

There was a direct but weak relationship between *OSCAR* gene and surfactant protein D level (r = 0.234 and *p* = .034), whereas there was an inverse and moderate relation between forced expiratory ratio FEV1/FVC and OSCAR gene expression (r = −0.630 and *p* = < .001).

There was a negative correlation of FEV1, FVC, and FEF2575 with OSCAR, as shown in Table 3. The relation between disease severity and *OSCAR* gene expression was significant r (79) = .51, *p* = < .001).

The regression analysis was used to see whether the gene expression has a certain level when linked with lung functions and SPD in the presence of age, BMI, and smoking exposure. It was observed that in the full model, age, FEV1, and FVC were found to have a significant effect on gene expressions as shown in Table 4, with *p*-values of 0.035, <0.001, and 0.001, respectively. Then, a reduced model was applied by eliminating insignificant variables, and the adjusted R2 increased to 0.266 as compared to 0.244 for the full model. Age had a negative impact, with a *p*-value of 0.020 and a coefficient of −2.86, explaining that gene expression decreases by 2.86-fold with each year increase after adjusting for low age in the control group. FEV1 had a coefficient of −146.18, indicating that each liter increase in FEV1 may reduce the gene expression by approximately 146-fold, while a per liter increase in FVC may increase the gene expression by 93.88-fold.

**TABLE 4:** Multiple linear regression for genetic expression of OSCAR and different variables.

	Full model (adjusted R^2^ = 0.241)			Reduced model (adjusted R^2^ = 0.266)		
Variable	β	*p*-value	Confidence interval	β	*p*-value	Confidence interval
Constant	255.77	0.044	7.42 to 504.12	249.79	0.007	68.86 to 430.72
Age (years)	−2.85	0.035	−5.49 to −0.20	−2.86	0.020	−5.43 to −0.46
BMI	0.35	0.909	−5.69 to 6.38			
Smoking (pack years)	−0.42	0.610	−2.03 to 1.20			
SPD	−1.14	0.734	−7.81 to 5.52			
FEV1 (L)	−155.31	<0.001	−219.49 to −91.14	−146.18	<0.001	−202.13 to −90.23
FVC (L)	99.24	0.001	42.09 to 156.39	93.88	0.001	40.85 to 146.90

	Full model (adjusted R^2^ = 0.220)			Reduced model (adjusted R^2^ = 0.230)		
Variable	Β	*p*-value	Confidence interval	β	*p*-value	Confidence interval
Constant	120.048	0.255	−88.600 to 328.695	15.878	0.295	−14.067 to 45.823
Age (years)	−1.356	0.266	−3.764 to 1.052			
BMI	−0.953	0.753	−6.976 to 5.070			
Smoking (pack years)	−0.687	0.424	−2.389 to 1.015			
SPD	−1.262	0.711	−8.014 to 5.491			
COPD	133.742	0.000	77.234 to 190.250	106.820	0.000	64.470 to 149.169

Dependent variable: genetic expression of OSCAR.

The second regression analysis was performed by taking COPD status as a variable and removing lung functions (as COPD status is determined based on lung functions). Here, in the full model, only COPD appeared to have a significant impact on gene expressions, showing all other factors like age, BMI, smoking status, and SPD as insignificant with an adjusted R2 of 0.220, and the reduced model included only COPD status as a variable with an adjusted R2 0.230. So, here, it can be predicted that those with COPD are supposed to have 106.8-fold higher gene expression than those without COPD, even if not considered for age, BMI, smoking, or SPD (Table 4).

## 5 Discussion

COPD, being a heterogeneous disease with a multifactorial origin, diverse progression, and variation in structural manifestation, clinical presentation, and treatment response, poses a challenge to clinicians and health authorities alike.

Recent endeavors have been directed toward identifying and measuring certain biomarkers that, alone or in concert with spirometry or other inflammatory chemokines, would be helpful in diagnosing and predicting the course of disease progression in patients with COPD. The relationship between biomarkers and lung functions (FEV1, FVC, and FEV1/FVC) has been extensively studied with promising results. Interestingly, SP-D is one such lung-specific biomarker that has not been fully investigated. SP-D, along with many other candidates like interleukin-6 (IL-6), club cell 16 (CC16), serum soluble receptor for advanced glycation end products (sRAGE), C reactive protein (CRP), and fibrinogen, has been studied to a great extent in COPD patients for disease presentation, progression, and treatment results (Stockley et al., 2019).

SP-D, owing to its lung specificity, easy assay in blood and sputum, and inherent functional and biological properties signifying a crucial role in the pathogenesis of COPD, appears to be a candidate in the quest for substantiating it as a disease marker of COPD. Obeidat et al. in their genome-wide association study (GWAS), established the protective role of SP-D on risk of COPD and enhanced decline in FEV1 (Obeidat et al., 2017). Keeping in view the important role of SP-D in COPD, plasma SP-D levels in patients with stable COPD and healthy controls were explored.

An increased (although non-significant) trend in plasma levels of SP-D in patients with COPD compared to controls was observed, as reported by Lv et al. (2022). Smoking status was also not significantly related to levels of SP-D. Serum SP-D concentrations of the COPD group were higher than in the control group. The patients in the current study had stable COPD status compared to those with COPD exacerbations in other studies. It might explain the non-significant increase in plasma SP-D levels, in line with the results of a non-significant relationship between SP-D and smokers with and without COPD, as shown by Andreeva et al. (2021). Liao published similar findings showing higher SP-D levels with no significant relationship in patients with incident COPD compared to those without the disease. In the current study, lung functions were significantly deteriorated in COPD patients as compared to controls, but the association of lung functions with SP-D appeared non-significant, similar to that proposed by Liao et al. (2021). Comparable trends, i.e., higher SP-D levels and deteriorating lung functions in COPD patients as compared to controls, were also observed in a similar study (Çiftci EŞ et al., 2018). Such findings suggest that SP-D plays an important role in maintaining surfactant hemostasis and possesses antioxidant, anti-inflammatory, and immunologic functions.

*In vitro* results of the current studies show that SP-D regulates the activities of immune cells, epithelial cells, fibrocytes, and smooth muscle cells. It has been shown that blood monocytes have chemokine and adhesion receptors, which allow them to migrate from the circulation to areas of infection or injury. Once on site, activated monocytes secrete pro-inflammatory cytokines such as interleukin 1 (IL-1), IL-6, tumor necrosis factor (TNF), and other inflammatory mediators, which orchestrate the recruitment of additional immune cell types. Monocytes differentiate into macrophages or dendritic cells depending on the local milieu (Kapellos et al., 2019).

Distinctive cell clusters of peripheral blood mononuclear cells (PBMCs) derived from COPD patients and healthy controls were identified, suggesting that impaired immune function may be linked to the progression of COPD (Pei et al., 2022). Increased OSCAR expression on monocytes is associated with disease activity in rheumatoid arthritis (RA) patients. As a costimulatory receptor, OSCAR promotes the production of osteoclasts from precursor monocytes. RA patients with active illness have monocytes with an increased expression of OSCAR (Schultz et al., 2016). In humans, OSCAR is reported to be expressed in monocytes, macrophages, neutrophils, and dendritic cells. The presence of OSCAR on human inflammatory cells leads to an investigation to elucidate its role in innate immunity, and thus, Barrow et al. (2015)identified SP-D as a candidate ligand of OSCAR and suggested that their interaction stimulated TNF-α production from monocytes (Barrow et al., 2015). Human OSCAR enhances the pro-inflammatory response of monocytes to develop into monocyte-derived macrophages (Belchamber et al., 2020). OSCAR can functionally interact with SP-D in inflammatory C-C chemokine receptor type 2 positive (CCR2+) monocytes and macrophages, resulting in a pro-inflammatory response. TNF-α is released from CCR2+ monocytes as a result of this interaction, and the SP-D/OSCAR complex appears to be internalized in alveolar macrophages, which orchestrate innate and adaptive immune responses in the lungs following lung injury by smoke or pollutants (Barrow et al., 2015; Watson et al., 2020). Interestingly, the findings of this study suggest a strong relationship between plasma SP-D levels and OSCAR gene expression, which partially explains the activation of blood monocytes and pulmonary interstitial macrophages. OSCAR gene expression was significantly increased in COPD patients and positively correlated with serum SP-D levels. Lung functions (FEV1, FVC, FEV1/FVC, and FEF2575) also showed a negative correlation with OSCAR gene expression. It is possible that SP-D released in airway lung surfactants would not be encountered by CCR2+ monocytes that patrol lung interstitial tissues in a healthy state. On the other hand, in response to pro-inflammatory or chemotactic stimuli like SP-D, monocytes gradually penetrate the airways. These monocytes replenish the alveolar macrophages and cause an increase in pro-inflammatory macrophages (M1) in comparison to anti-inflammatory macrophages (M2) in COPD, leading to worsening of pulmonary inflammation and subsequent deterioration of lung functions (Wu et al., 2022). This study is one of the first to show a positive correlation between gene expression of OSCAR and serum SP-D levels and a negative correlation between OSCAR gene expression and lung functions in stable COPD patients, hinting toward a more detailed role of SP-D in COPD disease pathogenesis and progression.

There were certain limitations to this work. First, it was a cross-sectional, single-center study with a limited number of samples and participants being exclusively men. The collection of blood for SP-D analysis and genetic expression of *OSCAR* was performed at a single point in time due to limitations in patient cooperation and follow-up. In the next phases, a prospective cohort study with random or systematic sampling of COPD patients and controls could be done to authenticate the findings of this study. Furthermore, the relationship between SP-D and inflammatory markers, especially TNF-α and ILs, can be investigated. Patients could be followed up to compare plasma SP-D levels and OSCAR gene expression in exacerbation and stable phases of COPD in a multicenter and large-sample study. Second, age could not be matched due to convenience sampling; although it was adjusted with multiple regression analysis, more accurate sampling techniques could be employed to minimize the selection bias. Third, all the patients were of the same ethnicity, due to which the data cannot be generalized to other populations, as the ethnicity may affect serum SP-D levels, lung functions, and gene expression. Fourth, SP-D and genetic expression were measured in stable COPD patients and controls without including patients having exacerbation of COPD. If a significant number of exacerbation patients were included and checked for the serum levels and relation between SP-D and gene expression of OSCAR, it might have provided an insight into the link between the two with greater evidence and confidence.

## 6 Conclusion

COPD patients exhibit a variety of clinical manifestations, treatment outcomes, exercise stamina, exacerbation risk, and airway damage. In the quest for a prognostic, diagnostic, or predictive biomarker, SP-D and OSCAR show the potential to impact disease diagnosis and patient care and reduce the burden on the healthcare system. The role of *OSCAR* in bone metabolism is well-established. This study found elevated *OSCAR* gene expression in the blood of COPD patients in the absence of any bone disease, like rheumatoid arthritis or osteoarthritis. It can aid in the early diagnosis of disease, identification of persons at risk, stratification of individuals on the basis of severity, monitoring of disease progression, and treatment. By validating the integrated relationship between SP-D and *OSCAR*, knowledge on the pathogenesis of COPD can be improved. New precision therapies and medications can be developed to modulate the activity of SP-D and *OSCAR*, thereby helping reduce pulmonary and systemic inflammation.

## Data Availability

The raw data supporting the conclusions of this article will be made available by the authors, without undue reservation.

## In memoriam

I would like to express my heartfelt gratitude to my late mentor and teacher, Professor Dr. Tania A. Shakoori, from the Physiology Department at the University of Health Sciences, Lahore, Pakistan. Her guidance, support, and unwavering dedication to science have been an immense source of inspiration for me. Her passion for teaching and research has profoundly influenced my work and career. This acknowledgment is a humble tribute to her memory and the lasting impact she has had on my professional journey.
